# Human mesenchymal stromal cells inhibit platelet activation and aggregation involving CD73-converted adenosine

**DOI:** 10.1186/s13287-018-0936-8

**Published:** 2018-07-04

**Authors:** P. Netsch, S. Elvers-Hornung, S. Uhlig, H. Klüter, V. Huck, F. Kirschhöfer, G. Brenner-Weiß, K. Janetzko, H. Solz, P. Wuchter, P. Bugert, K. Bieback

**Affiliations:** 1 Institute of Transfusion Medicine and Immunology, Medical Faculty Mannheim, Heidelberg University, German Red Cross Blood Donor Service Baden-Württemberg—Hessen, Friedrich-Ebert Straße 107, 68167 Mannheim, Germany; 2 Flow Core Mannheim, Medical Faculty Mannheim, Heidelberg University, Mannheim, Germany; 3 Center for Internal Medicine, University Medical Center Hamburg–Eppendorf, Hamburg, Germany; 4 Experimental Dermatology, Medical Faculty Mannheim, Heidelberg University, Mannheim, Germany; 5 Institute of Functional Interfaces, Karlsruhe Institute of Technology, Eggenstein-Leopoldshafen, Germany; 6 Institute for Clinical Chemistry, Medical Faculty Mannheim, Heidelberg University, Mannheim, Germany; 7 Mannheim Clinic for Plastic Surgery, Mannheim, Germany

**Keywords:** Mesenchymal stromal cells, Platelet activation, CD73, Adenosine, Ectonucleotidase activity, Flow cytometry

## Abstract

**Background:**

Mesenchymal stromal cells (MSCs) are promising cell therapy candidates. Clinical application is considered safe. However, minor side effects have included thromboembolism and instant blood-mediated inflammatory reactions suggesting an effect of MSC infusion on hemostasis. Previous studies focusing on plasmatic coagulation as a secondary hemostasis step detected both procoagulatory and anticoagulatory activities of MSCs. We now focus on primary hemostasis and analyzed whether MSCs can promote or inhibit platelet activation.

**Methods:**

Effects of MSCs and MSC supernatant on platelet activation and function were studied using flow cytometry and further platelet function analyses. MSCs from bone marrow (BM), lipoaspirate (LA) and cord blood (CB) were compared to human umbilical vein endothelial cells or HeLa tumor cells as inhibitory or activating cells, respectively.

**Results:**

BM-MSCs and LA-MSCs inhibited activation and aggregation of stimulated platelets independent of the agonist used. This inhibitory effect was confirmed in diagnostic point-of-care platelet function analyses in platelet-rich plasma and whole blood. Using inhibitors of the CD39–CD73–adenosine axis, we showed that adenosine produced by CD73 ectonucleotidase activity was largely responsible for the LA-MSC and BM-MSC platelet inhibitory action. With CB-MSCs, batch-dependent responses were obvious, with some batches exerting inhibition and others lacking this effect.

**Conclusions:**

Studies focusing on plasmatic coagulation suggested both procoagulatory and anticoagulatory activities of MSCs. We now show that MSCs can, dependent on their tissue origin, inhibit platelet activation involving adenosine converted from adenosine monophosphate by CD73 ectonucleotidase activity. These data may have strong implications for safety and risk/benefit assessment regarding MSCs from different tissue sources and may help to explain the tissue protective mode of action of MSCs. The adenosinergic pathway emerges as a key mechanism by which MSCs exert hemostatic and immunomodulatory functions.

**Electronic supplementary material:**

The online version of this article (10.1186/s13287-018-0936-8) contains supplementary material, which is available to authorized users.

## Background

Due to their numerous and promising therapeutic capacities, mesenchymal stromal cells (MSCs) are already applied clinically [1]. So far, clinical trials have documented the safety of MSC applications with rare and minor adverse events in humans [2–4]. In animals, however, increased death rates have been observed due to thromboembolic events after infusion [5–9]. Importantly, there has been one report of a patient dying following a pulmonary embolism, potentially related to the MSC application [10].

Thromboembolic events may occur via different mechanisms. First, the relatively large MSCs may be entrapped in and may subsequently occlude small vessels, particularly lung capillaries [7, 9]. This may explain why in animals the cell dose and infusion velocity have been linked to embolic side effects [11]. Second, MSCs may impact hemostasis and actively promote coagulation through high expression of procoagulant molecules like tissue factor (TF), triggering the clotting cascade [6, 12–14]. In consequence, clinical trial protocols have already been modified to add antithrombotics such as heparin or hirudin [12, 13, 15–17]. A recent study suggests selecting for TF-negative MSCs to avoid thromboembolism and IBMIR [14].

With respect to their procoagulant properties, MSCs from different tissue sources seem to differ at both expressional as well as functional levels [14, 18, 19]. In a mouse model, lipoaspirate-derived MSCs (LA-MSCs) were associated with higher risks of embolic events in comparison to bone marrow-derived MSCs (BM-MSCs) [9]. MSCs derived from placental decidua showed increased activation of plasmatic clotting compared to BM-MSCs [13].

In contrast, very few studies have considered the cellular component of thrombosis/hemostasis. Observed effects have varied from increased platelet thrombus formation [12, 15, 16] to antithrombotic properties in vascular grafts [20, 21]. We have therefore analyzed the influence of MSCs on platelet activation. We compared MSCs from different tissue sources (BM, LA and cord blood), and evaluated whether conditioned medium or cells stimulate or inhibit the activation of resting or agonist-induced activated platelets. Platelet activation and aggregation was measured using different methods including diagnostic point-of-care techniques.

## Methods

### Blood collection and preparation

Blood was collected with 21-gauge butterfly needles from antecubital veins into citrate phosphate dextrose adenine (CPDA)-containing or hirudin-coated tubes. Donors were volunteer healthy persons giving informed consent, who had not been taking any platelet inhibiting medication for at least 2 weeks.

Platelets were deployed for experiments immediately after collection. Either whole blood or platelet-rich plasma (PRP) was used, obtained by centrifugation of whole blood at 100 × *g* for 10 min. The PRP was diluted 1:1 with phosphate buffered saline (PBS) before subsequent use.

### MSCs, HUVECs and HeLa cells

Human MSCs from the three different tissue sources—bone marrow (BM), lipoaspirate (LA) and cord blood (CB)—as well as human umbilical vein endothelial cells (HUVECs) were isolated from multiple different donors and characterized as described previously [22, 23]. Experiments were approved by the Mannheim Ethics Commission II (vote numbers 2010-262 N-MA, 2009-210 N-MA, 49/05 and 48/05). HeLa tumor cells were kindly provided by Prof. Ilse Hofmann, DKFZ, Heidelberg, Germany. HUVECs and HeLa tumor cells served as controls; endothelial cells have been shown to inhibit and tumor cells to promote platelet activation [24, 25].

All cells were stored cryopreserved in fetal bovine serum (FBS)/10% DMSO and were then thawed and cultivated for at least one passage before use. HUVECs were cultured in EGM-2 (Lonza, Basel, Switzerland), and MSCs and HeLa cells in DMEM (Lonza) supplemented with 10% FBS (PromoCell, Heidelberg, Germany), 4 mM glutamine and antibiotics. To standardize conditions for MSCs, HUVECs and HeLa cells, respectively, cells were seeded at a defined density in T175 flasks 2 days before performing the experiments: MSCs at 1 × 10^6^ cells, passages 3–4 (to test for replicative aging also until passage 6); HUVECs at 2 × 10^6^ cells, passages 3–5; and HeLa cells at 5 × 10^6^ cells. Immediately before the experiments, the cells were detached with trypsin–EDTA, washed, counted and resuspended in PBS. The cell doses (10^5^, 5 × 10^5^, 2.5 × 10^6^ cells/ml) employed for our study were calculated according to the cell numbers applied clinically [1]. Conditioned medium (CM) was collected 48 h after seeding 10^6^ cells in T175 flasks. Pure culture medium served as a control.

### Flow cytometry

Flow cytometry was performed on a BD FACSCanto™ II (Becton Dickinson, Heidelberg, Germany). Data were obtained with BD FACS Diva software and analyzed with FlowJo software (FlowJo, LLC, Ashland, OR, USA).

Before stimulation, platelets were incubated at room temperature with respective cells or CM for 10 min in the presence of the staining antibodies. Following this, platelets were activated with TRAP-6 (protease-activated receptor 1 (PAR-1) agonist), ADP (P2Y1, P2Y12 and P2X1 receptor agonist) or U46619 (thromboxane A2 (TP) receptor agonist) (all 5 μM; Roche, Mannheim, Germany) for 10 min. Experiments were performed at staggered times or samples were fixed directly after the stimulation period by 0.5% paraformaldehyde and then analyzed.

Platelets were stained with an antibody panel including the activation-dependent markers PAC-1-FITC (activated GPIIb/IIIa (CD41/CD61) complex, clone PAC-1; Becton Dickinson), CD62P-APC (P-selectin, clone AK-4; Becton Dickinson) and CD63-PE-Cy7 (LAMP-3, clone H5C6; Becton Dickinson) and the platelet-specific surface marker CD41-PE (IIb subunit of GPIIb/IIIa complex, clone HIP8; Beckman Coulter, Krefeld, Germany) [26]. Antibodies had been titrated. A platelet gate was set according to FSC-PE and at least 10,000 events were recorded.

To quantify the respective receptor expression by flow cytometry, cells were stained with anti-CD73 (PE, clone AD-2; Becton Dickinson), anti-CD39 (PerCP-Cy5.5, clone TU66; Becton Dickinson) and anti-adenosine A2A receptor (A2AR, 7F6-G5-A2 Alexa Fluor® 488; Santa Cruz Biotechnology, Heidelberg, Germany).

### Inhibitors

Different mechanisms have been shown to interfere with platelet activation. To understand which is affected by MSCs, we used different inhibitors, as specified in the following [27–29].

CD62P was blocked by the mouse anti-human antibody AK-4 (eBioscience, ThermoFisher, San Diego, CA, USA). PRP 50 μl was preincubated with 1 μg AK-4 or the respective isotype control for 20 min before adding the MSCs.

For COX inhibition, MSCs were cultured with indomethacin (10 μM; Sigma Aldrich) for 2 days. Two hours before the experiments the medium was changed and fresh indomethacin added to the coculture.

CD39 was inhibited by the antagonist sodium polyoxotungstate (100 and 10 μM POM-1; Tocris, Wiesbaden-Nordenstadt, Germany), CD73 inhibited by AMP-CP (100 μM α,β-methyleneadenosine 5′-diphosphate; Santa Cruz), adenosine receptors inhibited by caffeine (200 μM; Santa Cruz) and A2A adenosine receptor was inhibited by 25 μM SCH 58261 (Tocris, 25 mM stock in DMSO, DMSO used as control). Alkaline phosphatase (ALP) was inhibited by levamisole hydrochloride (100 μM; Abcam, Cambridge, UK). Adenosine deaminase (ADA) from calf intestine (2.55 U/ml; Sigma Aldrich) was added to deaminate adenosine [30, 31]. All inhibitors were preincubated for 10 min with MSCs before adding PRP. Adenosine (1 μM; Santa Cruz) was used as positive control. For most inhibitors, different dilutions have been tested to identify the working concentration.

### Detection of ectonucleotidase activity

Ectonucleotidase activity was measured in the cells as described previously [32]. Briefly, cells were seeded at 10,000 cells/cm^2^ in 24-well plates and then incubated for 24 h. ATP, ADP and AMP (1 mM; Santa Cruz) were then added in phosphate-free buffer and incubated for either 1 h (ATP and ADP) or 30 min (AMP). Supernatant was harvested for protein quantification (BCA assay; Thermo Fisher, Waltham, MA, USA), inorganic phosphate quantification (malachite green assay kit, according to the manufacturer’s instructions; Sigma Aldrich) or adenosine detection.

### Quantitative determination of adenosine by LC-MS/MS

The samples were separated by HPLC (Agilent 1100, Waldbronn, Germany) using a LiChrospher 100 RP C-18, 5 μm column (125 mm × 4 mm) in combination with a gradient method of acetonitrile and 0.1% acetic acid at a flow rate of 500 μl/min. Mass spectrometric analysis was carried out using an API 4000™ quadrupole mass spectrometer (Applied Biosystems/MDS Sciex, Toronto, Canada) equipped with an electrospray ionization (ESI) source in the positive mode. MS/MS infusion experiments were performed to determine the specific mass transitions of adenosine (quantifier m/z 268 to m/z 136, qualifiers m/z 268 to m/z 119 and m/z 268 to m/z 92) for multiple reaction monitoring (MRM) analysis. All quantitative analyses were carried out using a sample volume of 10 μl containing adenosine-d5 as internal standard. The adenosine content of the samples was determined by a standard calibration function in the required concentration range.

### Alkaline phosphatase and adenosine deaminase activity measurement

Both enzymatic activities were measured in cell lysates with defined cell numbers by fluorometric assay kits according to the manufacturer’s instructions (ALP assay kit ab83371 and ADA assay kit ab204695; both Abcam).

### Vasodilator-stimulated phosphoprotein phosphorylation state

The phosphorylation state of vasodilator-stimulated phosphoprotein (VASP), indicative for cyclic nucleotide levels in platelets, was measured using cytometric bead technology (VASPFix; Platelet Solutions, Nottingham, UK) [33]. Briefly, PRP was coincubated for 10 min with 10 μM adenosine or 5 × 10^5^ cells/ml, followed by addition of ADP (5 μM). After 5 min, 5 μl of the platelet suspension was mixed with 25 μl of VASPFix reagent, vortexed and incubated for 2 h. The VASP phosphorylation state was assessed by flow cytometry using an APC (bead) and FITC (VASP-P) dot-plot gate, assessing the change in VASP-P-FITC mean fluorescence intensity (MFI).

### Platelet function analyzer

Platelet adhesion, activation and aggregation were assessed in a system simulating the in-vivo hemodynamics in the small capillaries (PFA-100; Siemens Healthcare Diagnostics, Eschborn, Germany). Citrated whole blood was aspirated at high shear rates though a small aperture coated with collagen and ADP. The gradual occlusion of the aperture by adhering platelets was measured as the closure time. Briefly, 900 μl of citrate-anticoagulated whole blood (platelet count > 150,000/μl and hematocrit > 35%) was mixed with either 10 μM adenosine or 5 × 10^5^ cells/ml and incubated for 10 min. The whole blood suspension (800 μl) was added to the analysis cuvettes, the ADP/collagen measurement was started and the closure time recorded.

### Light transmission aggregometry

The rate and extent of platelet activation, aggregation and agglutination was measured by light transmission aggregometry. PRP was stirred in a cuvette at 37 °C and photometrically monitored. Agonist-induced activation and aggregation induces a change from light absorbance to increased transmission (Platelet Aggregation Profiler®, Model PAP-8E; MöLab GmbH, Langenfeld, Germany). Briefly, PRP was prepared by centrifugation at 150 × *g* for 10 min and then carefully removed. The remaining blood was centrifuged at 2700 × *g* for 15 min to obtain platelet-poor plasma (PPP). The PPP was used to calibrate the system to 100% light transmission. Measurements were made on 250 μl aliquots of PRP preincubated for 10 min with 10 μM adenosine or with 5 × 10^5^ cells/ml at 37 °C in the aggregometry cuvette, after addition of 5 μM ADP.

### Real-time quantitative PCR

Real-time quantitative PCR (RT-qPCR) of procoagulant and anticoagulant factors was performed as described previously [34]. The RNeasy Mini Kit® (Qiagen, Hilden, Germany) was used for mRNA isolation, the Transcriptor High Fidelity cDNA Synthesis Kit (Roche Diagnostics, Mannheim, Germany) for cDNA transcription and the SensiFast™ Probe No-ROX Kit (Bioline, Luckenwalde, Germany) for PCR. The intron-spanning primers and probes (Universal Probe Library, Roche, Mannheim, Germany) presented in Additional file 1: Table S1 were used with a Light Cycler 480 (Roche). Relative quantification was performed using the Ε-method with GAPDH and SFRS4 as reference genes. The efficiency of all primers was in the range of 1.9–2.2.

### Statistical analysis

For statistical analysis, the degree of platelet activation was quantified by the mean fluorescence intensity (MFI) using flow cytometry. For some experiments, data were normalized to the respective control without cells/stimulator/inhibitor added. Significance testing was performed using a paired *t* test, repeated-measures one-way ANOVA, one-way or two-way ANOVA followed by Holmes–Sidak or Dunnett’s test or the Kruskal–Wallis test for nonparametric data (Sigma Plot 11.0; Systat Software, San Jose, CA, USA; and GraphPad Prism 7; GraphPad Software, La Jolla, USA).

## Results

### MSC conditioned medium does not affect platelet activation

**Fig. 1:**
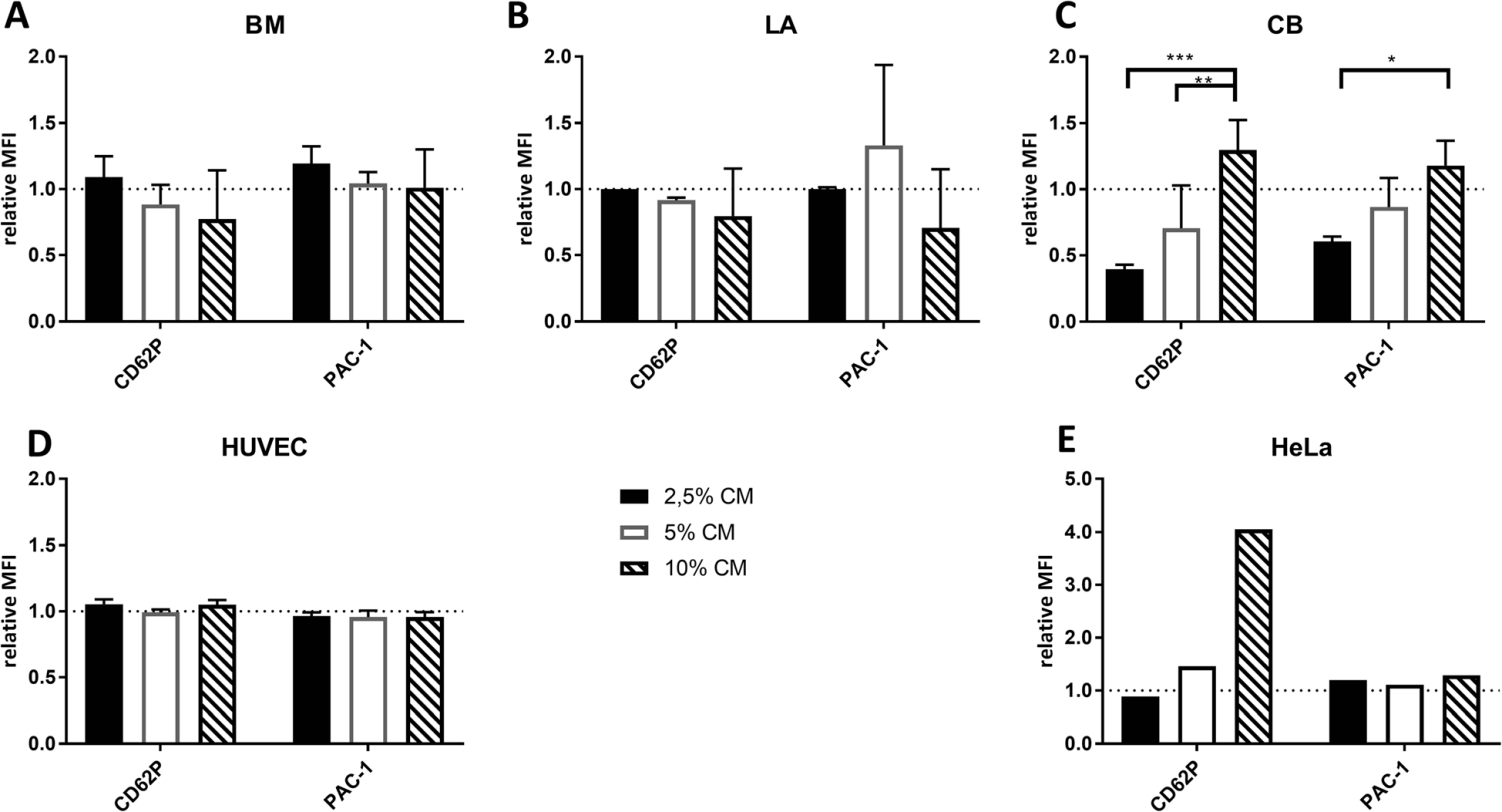
Effect of conditioned medium from different cell types on platelet activation. Platelets incubated with either cell culture or conditioned medium (CM) and then stimulated by TRAP-6. Platelet activation measured by flow cytometry, assessing expression of CD62P, CD63 and PAC-1 binding. **a** Bone marrow (BM)-MSCs. **b** Lipoaspirate (LA)-MSCs. **c** Cord blood (CB)-MSCs. **d** Human umbilical vein endothelial cells (HUVECs). **e** HeLa tumor cells. Scale indicates relative activation marker expression of CM compared to culture medium. *n* = 3 biological replicates; HeLa cells *n* = 1, note different *y* axis for HeLa cells. *p < 0.05, **p < 0.01, ***p < 0.001. MFI mean fluorescence intensity

To see whether MSCs produce any soluble factors influencing platelet activation, we quantified activation marker expression on resting (w/o agonist) and ADP or TRAP-6-stimulated platelets. There was no effect of any culture medium (CM) on resting platelets (data not shown). On agonist-stimulated platelets, there was also no effect of CM on BM-MSCs and LA-MSCs, independent of the added dose of 2.5–10% (Fig. 1). Low concentrations of CB-MSC CM suppressed the activation of platelets (0.40 ± 0.034 for CD62p and 0.61 ± 0.036 for PAC-1 at 2.5% CM compared to the control set to 1), but higher concentrations did not suppress compared to the control (10% CM, 1.30 ± 0.23 for CD62p and 1.18 ± 0.19 for PAC-1; significant differences between 2.5 and 10% CM). HUVEC CM had no effect on platelet activation (1.05 ± 0.04 for CD62p and 0.96 ± 0.04 for PAC-1 at 10% CM). HeLa CM, however, increased the agonist-induced activation of platelets dose dependently (from 0.89-fold at 2.5% CM to 4.05-fold at 10% CM for CD62p).

### MSCs inhibit agonist-induced platelet activation

**Fig. 2:**
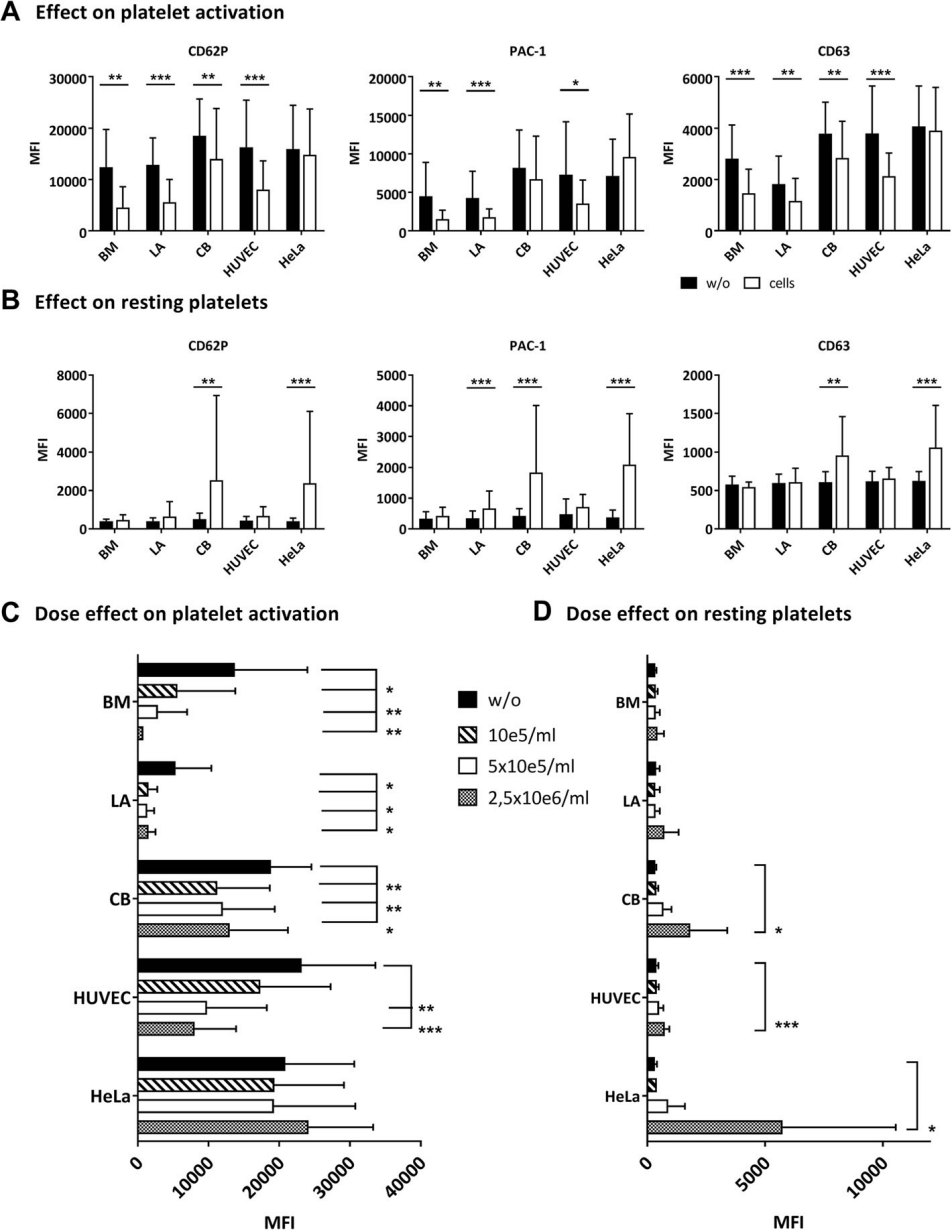
Effect of different cell types and dosages on platelet activation and resting platelets in flow cytometry. Platelets incubated with or without (w/o) indicated cell types. Then platelets either activated by TRAP-6 or remained resting. **a, b** Influence of 5 × 10^5^ cells/ml on **a** TRAP-6-induced platelet activation and **b** resting platelets. Mean fluorescence intensity (MFI) of different thrombocyte activation markers assessed: CD62P, PAC-1 binding and CD63 (*n* = 8–17). **c, d** Dose effect of different cell types on CD62P detection on **c** TRAP-6-stimulated platelets and **d** resting platelets (*n* = 5–6). Different MFI values of platelets explained by different platelet donors used in individual experiments. In all experiments, paired analysis against control w/o cells was performed. *p < 0.05, **p < 0.01, ***p < 0.001. BM bone marrow, CB cord blood, HUVEC human umbilical vein endothelial cell, LA lipoaspirate

Having observed that CM does not affect platelet activation, we then assessed the effect in cell–platelet cocultures. When platelets were agonist stimulated, all MSCs reduced the degree of platelet activation. All activation markers assessed were similarly affected (Fig. 2a). For CB-MSCs, however, donor-specific batch variation was apparent, with some batches even increasing platelet activation. HUVECs, as expected, reduced platelet activation, while HeLa cells had no significant effect. Next, we tested different MSC concentrations in the range of the concentrations employed in clinical treatments (10^5^, 5 × 10^5^, 2.5 × 10^6^ cells/ml). There was a clear dose-dependent effect of BM-MSCs (Fig. 2c for CD62p; other markers not shown). Interestingly, for LA-MSCs this was not apparent and with higher CB-MSC numbers the inhibitory effect was reduced, similar to the CM. HUVECs caused the expected dose-dependent inhibition, while HeLa cells had no significant effect at any concentration.

Next, we checked whether the MSCs activate resting platelets. BM-MSCs, LA-MSCs and HUVECs did not activate resting platelets (Fig. 2b, d). Interestingly, 2.5 × 10^6^ CB-MSCs/ml led to a 5-fold increase in CD62P expression and an 11-fold increase in PAC-1 binding, comparable to HeLa cells.

We verified that the inhibitory effect was donor specific and not affected by cellular aging (passages 3–6; data not shown), as reported for other MSC properties [35]. Different culture supplements used to expand MSCs (FBS, human AB serum or human platelet lysate) did not affect the inhibitory capacity of either BM-MSCs or LA-MSCs (data not shown) [36].

**Fig. 3:**
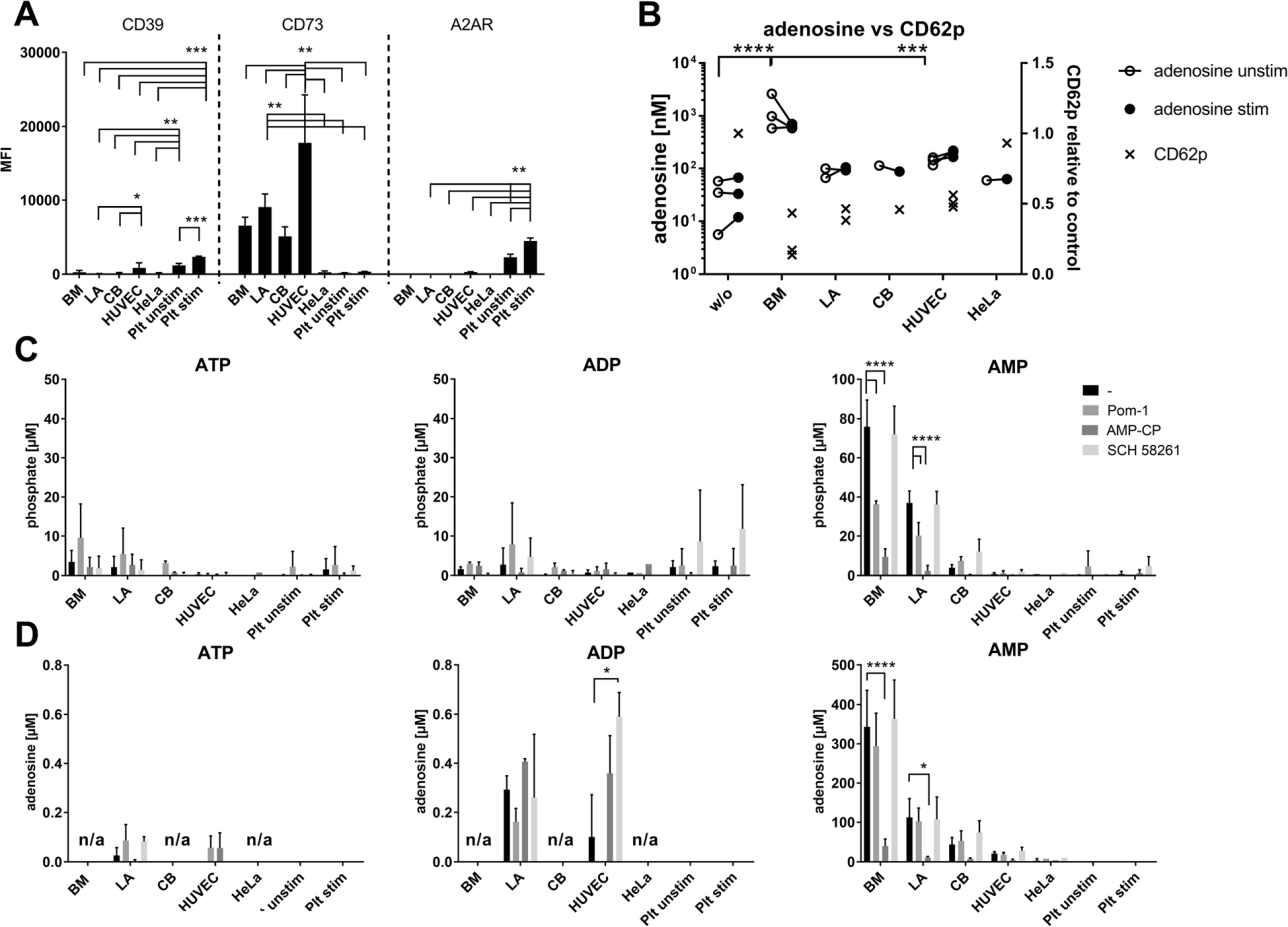
Expression and ectonucleotidase activity of CD39, CD73 and A2AR. **a** Cells stained with respective antibodies and MFI values determined by flow cytometry. BM-MSCs *n* = 3, LA-MSCs *n* = 7, CB-MSCs *n* = 5, HUVECs, *n* = 6 each biological replicates; HeLa *n* = 3 technical replicates; Plt unst, Plt stim each *n* = 4 biological replicates. *p < 0.05, **p < 0.01, ***p < 0.001. **b** Adenosine concentration (circles, left *y* axis) measured by mass spectrometry in supernatants of cell and platelet cocultures, either unstimulated or TRAP-6 stimulated. In parallel, inhibitory effect on TRAP-6-induced CD62p expression (crosses, right *y* axis) calculated relative to the TRAP-6 control. BM-MSCs *n* = 3, LA-MSCs *n* = 2, CB-MSCs *n* = 1, HUVECs *n* = 3 each biological replicates; HeLa *n* = 1. ***p < 0.001, ****p < 0.0001. **c, d** Released phosphate (**c**) or adenosine (**d**) indicative of ectonucleotidase activity determined after incubating cells with ATP, ADP and AMP in presence of respective inhibitors (CD39, POM-1; CD73, AMP-CP; A2AR, SCH 58261). BM-MSCs, LA-MSCs, CB-MSCs, HUVECs, Plt unst, Plt stim each *n* = 3 biological replicates; HeLa *n* = 1. *p < 0.05, ****p < 0.0001. Note different *y* axis scale for AMP. ADP adenosine diphosphate, AMP adenosine monophosphate, ATP adenosine triphosphate, BM bone marrow, CB cord blood, HUVEC human umbilical vein endothelial cell, LA lipoaspirate, n/a not analyzed, Plt stim stimulated platelets, Plt unst unstimulated platelets, w/o without

Additional experiments were performed: under shear-flow conditions, BM-MSCs likewise appeared to reduce the number of platelet aggregates formed on fibronectin (Additional file 2: Figure S1); using impedance aggregometry in whole blood, all cell types reduced platelet aggregation (multiplate device, Additional file 3: Figure S2); and potential platelet binding to MSCs was assessed by microscopy and flow cytometry. TRAP-6-stimulated platelets formed thrombi. HeLa cells and also CB-MSCs induced aggregation of activated and resting platelets (Additional file 4: Figure S3A–I, *p* = 0.004 for unstimulated vs stimulated platelets and *p* = 0.02 for unstimulated platelets vs stimulated platelets + HeLa cells), whereas BM-MSCs prevented stimulus-induced platelet aggregation (Additional file 4: Figure S3I). No platelet binding to any of the cells was apparent using flow cytometry, assessed by gating on MSC FSC/SSC and then calculating for CD41 positivity (Fig. 3J, no variance between cells w/o platelets and with unstimulated or stimulated platelets comparing *n* = 3 biological replicates for MSC and HUVECs, respectively).

### MSCs inhibit platelet activation independent of the activation pathway, P-selectin and cyclooxygenase

The fact that BM-MSCs and LA-MSCs significantly reduced platelet activation prompted the question of whether specific activation pathways are affected. Using flow cytometry, we measured three individual activation markers, CD62p, PAC-1 and CD63, plus three different platelet agonists, TRAP-6 (chosen for all subsequent analyses), ADP and U46619. Inhibition was apparent independent of the anticoagulant (data not shown) and for all three activation-dependent markers and agonists (Figs. 1 and 2, Additional file 5: Figure SA, B). Based on these data we conclude that the MSC-mediated effect is not directly linked to a specific pathway but interferes with platelet activation globally.

Endothelial progenitor cells have been shown to suppress platelet activation via CD62p and COX [27, 28]. However, neither the CD62P blocking antibody AK4 nor the nonselective COX inhibitor indomethacin were capable of neutralizing the MSC inhibitory activity (Additional file 5: Figure S4C, D), although MSCs express Cox-2 mRNA at highly differing levels (data not shown).

### CD73-converted adenosine is involved in MSC platelet inhibitory activity

We postulated that adenosine converted by CD73 ectonucleotidase activity may be responsible for the platelet inhibition. Adenosine has been shown previously to be inhibitory in endothelial–platelet interactions, and to contribute to MSC immunomodulatory activity [29, 37–40]. Extracellular ATP metabolism provides the prothrombotic ligands ATP and ADP (released from dense granula upon platelet activation and hydrolyzed by CD39) and the antithrombotic AMP and adenosine (by CD73 activity) detected by P1 adenosine receptors (on platelets provides mainly adenosine A2 receptor (A2AR)) [29, 41].

We first checked CD39, CD73 and A2AR expression. Platelets expressed both CD39 and A2AR at high MFI values, whereas CD73 was only dimly expressed (Fig. 3a). In MSCs, in contrast, CD73 was highly expressed, but CD39 and A2AR only dimly. HUVECs had the highest CD73 reactivity, with low CD39 and A2AR expression.

To test our hypothesis, we measured platelet activation and in parallel the adenosine concentration in MSC–platelet cocultures (Fig. 3b). In the presence of MSCs and HUVECs, adenosine levels were increased irrespective of TRAP-6 stimulation. HUVECs, despite a higher CD73 expression, had adenosine levels only slightly higher than LA-MSCs and CB-MSCs, with comparable antithrombotic activity. BM-MSCs, which showed the highest adenosine concentrations, exerted the highest inhibitory activity on platelet activation. These data support our hypothesis that MSC-generated adenosine conferred antithrombotic activities. In fact, the concentrations measured in cocultures were exactly in the range of the inhibitory adenosine concentration, strongest at 10–0.01 μM adenosine for both PRP and whole blood (see Fig. 6a).

To quantify the activity of CD39 and CD73, cells were incubated with ATP, ADP and AMP and with inhibitors of the nucleotide degradation cascade (POM-1 for CD39, AMP-CP for CD73 and SCH 58261 for A2AR). The released phosphate and adenosine was measured. ATP and ADP generated only minor amounts of phosphate and adenosine, indicating very low ATP/ADP degradative properties. However, MSCs were able to catabolize AMP to phosphate and adenosine (Fig. 3b, c), significantly inhibited by AMP-CP, indicating that CD73 activity is crucial for AMP conversion. Despite the high expression of CD73 in HUVECs, they showed little phosphate and adenosine production. In BM-MSCs and LA-MSCs, POM-1 significantly inhibited phosphate, but not adenosine generation, suggesting a minor involvement of CD39. Platelets per se, neither unstimulated nor stimulated, produced detectable amounts of adenosine.

**Fig. 4:**
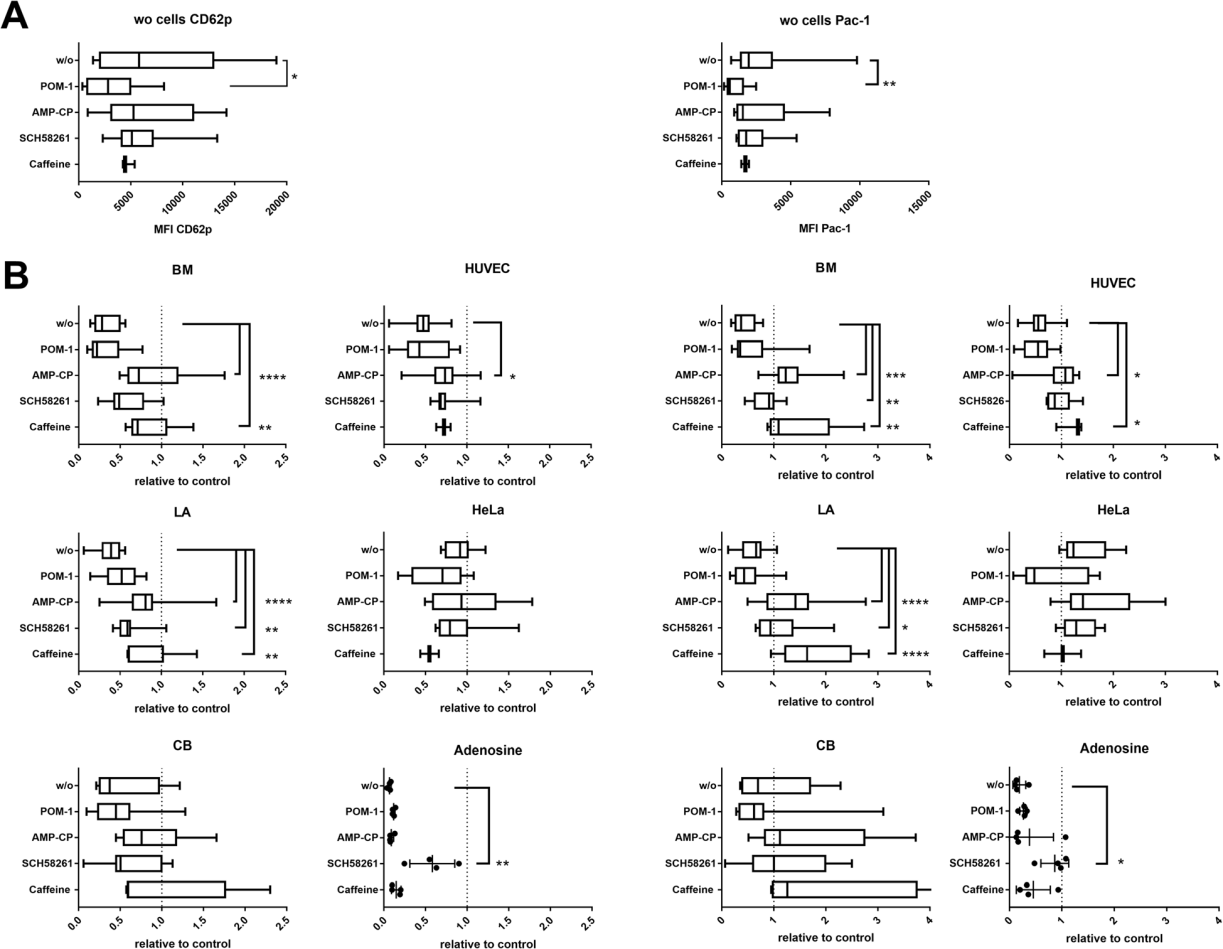
Blockade of different factors involved in nucleotide degradation cascade. Platelets incubated with cells and either POM-1 to block CD39, AMP-CP to block CD73, SCH58261 to specifically block adenosine receptor A2A or caffeine as nonspecific P1R inhibitor followed by TRAP-6 activation. Effects on CD62P expression (**a**) and PAC-1 binding (**b**) shown. **a** Effect of inhibitors on platelets alone (MFI of CD62p and PAC-1), *n* = 3–18 (*n* = 3 for caffeine). *p < 0.05. **b** Effects induced by adding inhibitors (CD39, POM-1; CD73, AMP-CP; A2AR, SCH 58261; nonspecific P1R, caffeine) in platelet–cell cocultures. Data normalized against respective control activated by TRAP-6 without cells (dotted line at value 1). BM-MSCs *n* = 6–11, LA-MSCs *n* = 5–21, CB-MSCs *n* = 5–9, HUVECs *n* = 3–12 biological replicates; HeLa cells *n* = 2–9, adenosine *n* = 4. *p < 0.05, **p < 0.01, ***p < 0.001, ****p < 0.0001. BM bone marrow, CB cord blood, HUVEC human umbilical vein endothelial cell, LA lipoaspirate, MFI mean fluorescence intensity, w/o without

To verify that CD73-converted adenosine regulates platelet reactivity, we added the aforementioned inhibitors to MSC–platelet cocultures. POM-1 (both 100 and 10 μM) significantly reduced TRAP-induced platelet activation (Fig. 4a; ADP and U46619 not shown), supporting our notion that CD73-mediated adenosine generation causes platelet inhibition (Fig. 4b) [42]. AMP-CP and SCH 58261 had no effect on platelet activation per se. POM-1 did not counteract the MSC-mediated antithrombotic activity. The CD73 inhibitor AMP-CP, however, significantly antagonized the inhibitory effect of BM-MSCs, LA-MSCs and HUVECs, supporting our notion that CD73-mediated adenosine generation causes platelet inhibition (Fig. 4c–f). In CB-MSCs, again, data varied with different cell batches. The adenosine receptor inhibitors, SCH 58261 specific for A2AR and nonspecific P1 receptor inhibitor caffeine, partially reversed the inhibitory effects of BM-MSCs and LA-MSCs, demonstrating that adenosine sensed by adenosine receptors converts the inhibitory signal. The facts that caffeine exerted a stronger effect in reducing MSC inhibition while SCH 58261 strongly reduced the inhibitory activity of adenosine suggest that A2AR is the main adenosine receptor on platelets, but that in MSC–platelet cocultures other P1 receptors are predominant, probably expressed on MSCs [43]. These findings indicate that the CD73–adenosine axis is a key mechanism in platelet inhibition by MSCs. It was striking that PAC-1 expression was strongly increased in the presence of the inhibitors exceeding the expression level of stimulated platelets (set to 1). This suggests that when the inhibitory adenosine action is inhibited, MSCs can accelerate induced platelet activation acting on specific pathways.

**Fig. 5:**
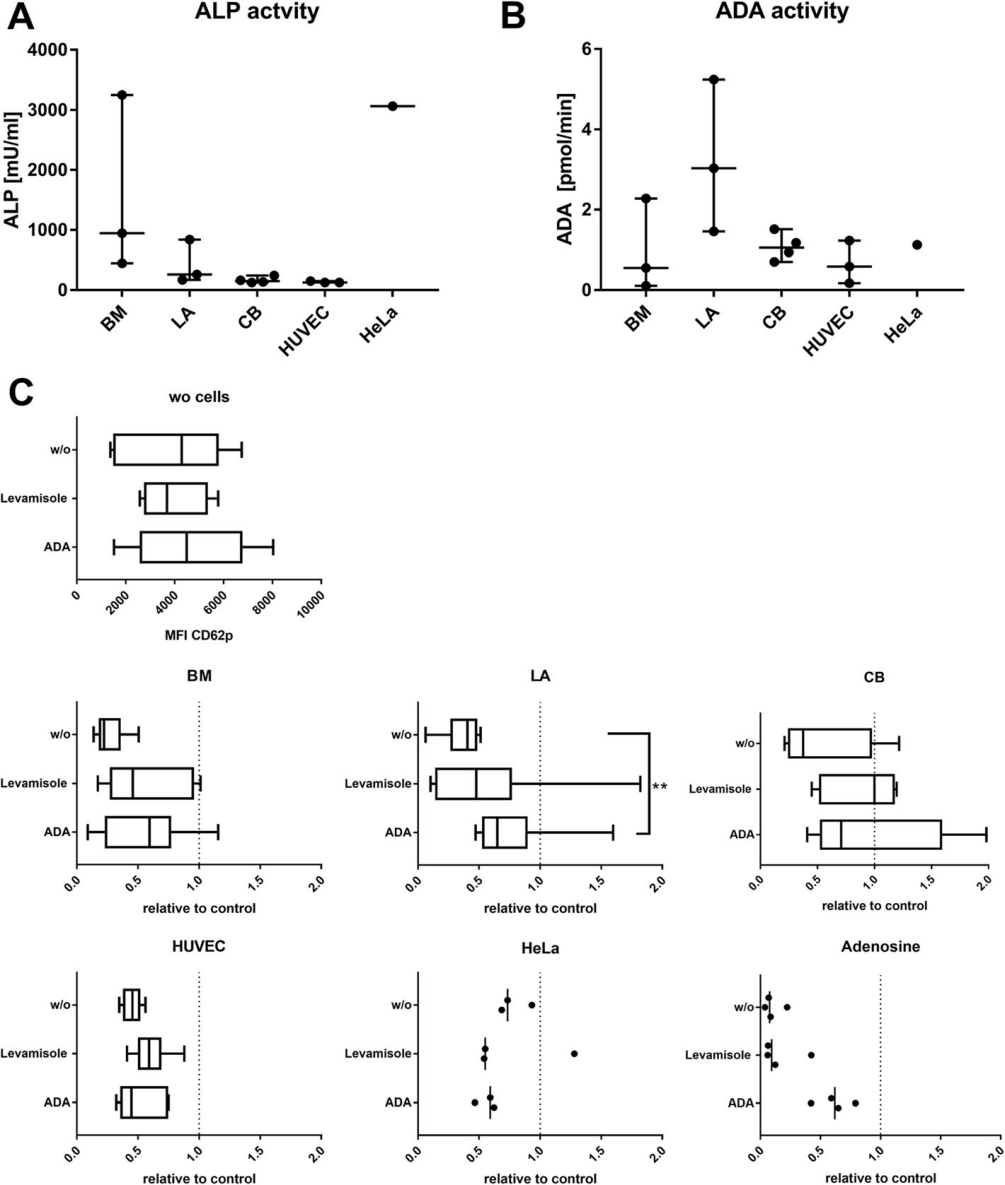
Alkaline phosphatase and adenosine deaminase activity and function blocking. **a** Alkaline phosphatase (ALP) and **b** adenosine deaminase (ADA) activity in different cell types. Individual biological replicates depicted as dots. **c** Effects induced by adding ALP inhibitor levamisole or ADA in platelet–cell cocultures. Data normalized against control activated by TRAP-6 without cells (dotted line at value 1). w/o *n* = 4–11, BM-MSCs *n* = 9, LA-MSCs *n* = 7–12, CB-MSCs *n* = 5–9, HUVECs *n* = 6 biological replicates; HeLa cells *n* = 3, adenosine *n* = 4. **p < 0.01. BM bone marrow, CB cord blood, HUVEC human umbilical vein endothelial cell, LA lipoaspirate, MFI mean fluorescence intensity, w/o without

For HUVECs and BM-MSCs there was a large discrepancy between CD73 expression intensity and enzymatic activity, suggesting that other factors are involved or that CD73 expression does not correlate to enzymatic activity. Alkaline phosphatase (ALP) can act synergistically to CD73 to metabolize AMP to adenosine [31]. Adenosine deaminase (ADA) activity converts adenosine to inosine, stopping the inhibitory action of adenosine [30]. HeLa cells showed high ALP activity (Fig. 5a). BM-MSCs exerted a highly batch-dependent ALP activity, while the ALP activity of LA-MSCs, CB-MSCs and HUVECs was low. ADA activity was comparable for all samples, except for LA-MSCs where two out of the three donors showed high ADA activity. To test for their effects in MSC–platelet cocultures, we added the ALP inhibitor levamisole and ADA. Levamisole at a concentration of 1 mM inhibited platelet activation per se, similar to POM-1. At 100 μM this inhibitory effect was negligible (Fig. 5c). Levamisole slightly, but not significantly, reduced the inhibitory effect of all MSCs and HUVECs. Externally added ADA ameliorated the adenosine action and significantly reduced the inhibitory effect of LA-MSCs, probably adding on the ADA activity of LA-MSCs to achieve adenosine neutralization.

### Verification using additional platelet function analyses

To document that MSCs triggered adenosine signaling in platelets, and that the suppressive strength was relevant not only in PRP but also in whole blood, we performed further platelet function tests including point-of-care technologies.

**Fig. 6:**
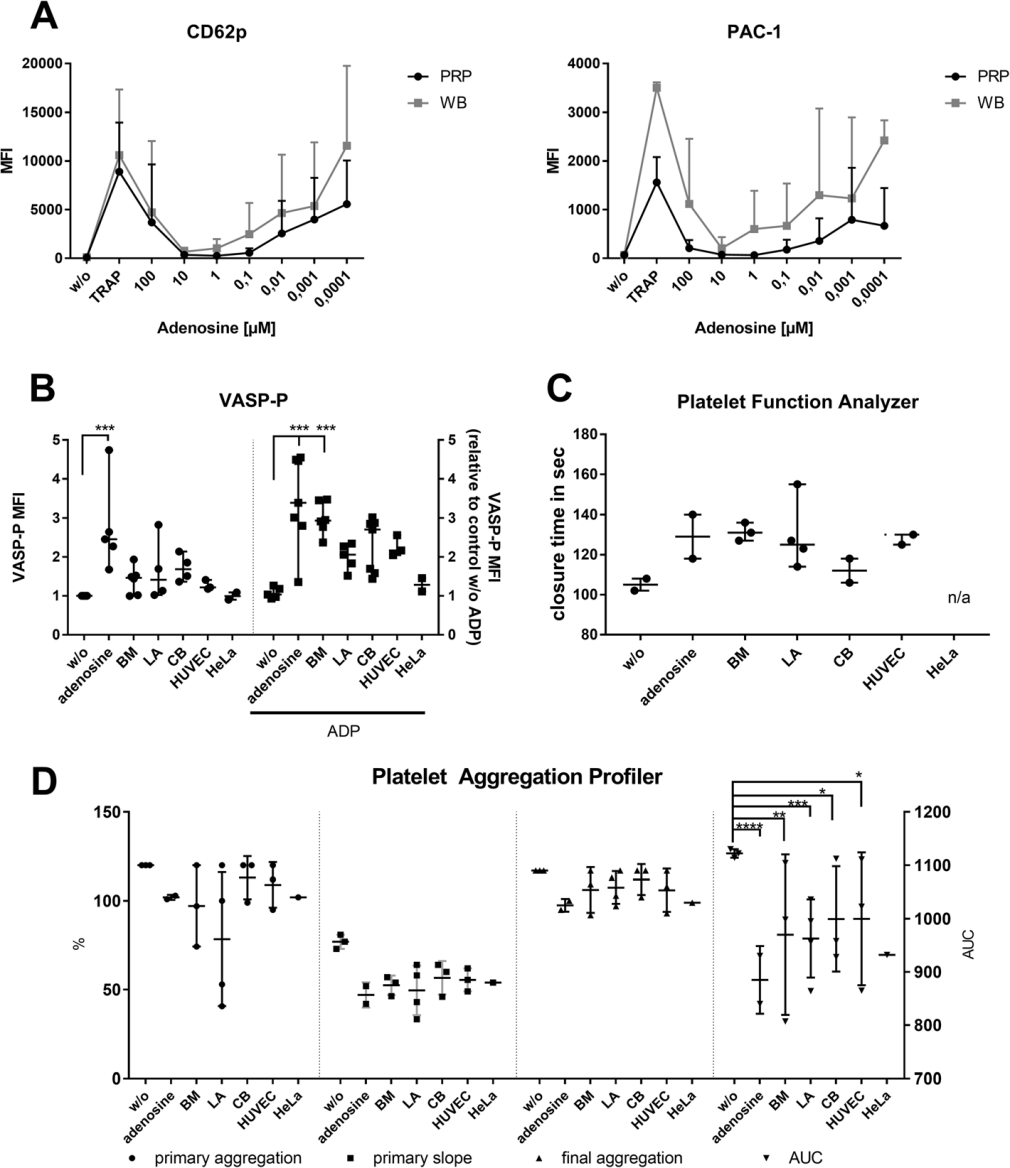
Platelet function analyses. Platelet function analyses. **a** Dose-dependent effect of adenosine on platelet activation (MFI of CD62p and PAC-1, n=3). **b** Phosporylation state of vasodilator-stimulated phoshoprotein (VASP), VASP-P MFI levels **c** Closure time measured as time platelets need to close an ADP/collagen-coated aperture measured using PFA-100 device, comparing nontreated, adenosine and cell-cocultured whole blood. ***p < 0.001. **d** Platelet function assessed using light transmission aggregometry (platelet aggregation profiler). Aggregation cascade can be separated into primary aggregation and slope, final aggregation (all left *y* axis) and area under the curve (AUC; right *y* axis) assessing entire aggregation response. *p ≤ 0.05, **p ≤ 0.01, ***p ≤ 0.001, ****p ≤ 0.0001. ADP adenosine diphosphate, BM bone marrow, CB cord blood, HUVEC human umbilical vein endothelial cell, LA lipoaspirate, MFI mean fluorescence intensity, n/a not analyzed, PRP platelet-rich plasma, TRAP thrombin receptor activator for peptide, WB whole blood, w/o without

First, we checked the concentration range of adenosine in PRP and whole blood. In fact, adenosine concentrations obtained in MSC–platelet cocultures were in the inhibitory range of adenosine in both PRP and whole blood (Fig. 6a).

Second, the phosphorylation state of vasodilator-stimulated phosphoprotein (VASP) was assessed in ADP-stimulated PRP. VASP-P levels are indicative of cAMP levels, known as master switches of platelet activation and aggregation [33, 41]. Adenosine activates adenylate cyclase, increasing cAMP levels and VASP phosphorylation, thus inhibiting platelet aggregation [44]. Indeed, adenosine increased the MFI of VASP-P, further raised by ADP stimulation (Fig. 6b). Without ADP stimulation, MSCs and HUVECs led to a slight increase and to a further rise upon ADP stimulation that was significant for BM-MSCs. CB-MSCs clearly split into two clusters, one inducing little VASP-P elevation after ADP addition while the other promoted VASP-P levels similar to BM-MSCs. HeLa cells did not influence VASP-P. These data support that MSCs induce the same signaling events as adenosine.

Third, we analyzed the MSC effects on platelet adhesion, activation and aggregation in whole blood using the platelet function analyzer (PFA-100), a well-established diagnostic point-of-care test [45]. As expected, adenosine prolonged the time needed to form a platelet plug closing an aperture (Fig. 6c). BM-MSCs, LA-MSCs and HUVECs likewise delayed platelet function, while CB-MSCs exerted a minor effect. HeLa cells repeatedly caused an error in the measurement.

Fourth, we used light transmission aggregometry (platelet aggregation profiler, PAP 8E), which distinguishes between the different stages of platelet activation: primary aggregation induced by the added agonist; secondary aggregation induced by endogenous agonists; and maximal and final aggregation which may differ when disintegration of platelet aggregates occurs. The speed of aggregation is measured as a primary slope. Area under the curve (AUC) values reflect the entire reaction cascade. Upon ADP stimulation, adenosine reduced the primary aggregation response, the speed and also the final aggregation compared to the control (Fig. 6d). The effects of BM-MSCs and LA-MSCs were similar. CB-MSCs and HUVECs exerted less pronounced platelet inhibition. HeLa cells (*n* = 1) showed a similar pattern, possibly related to the platelet donor (slight inhibitory activity was seen in the corresponding flow cytometry experiments). Despite the low sample number, AUC values indicated statistically significant differences of adenosine; all MSCs and HUVECs reduced platelet aggregation. In conclusion, all performed platelet function analyses confirmed the inhibitory effect of at least BM-MSCs and LA-MSCs on platelet function to a similar extent as adenosine.

### Gene expression of procoagulant and anticoagulant factors

**Fig. 7:**
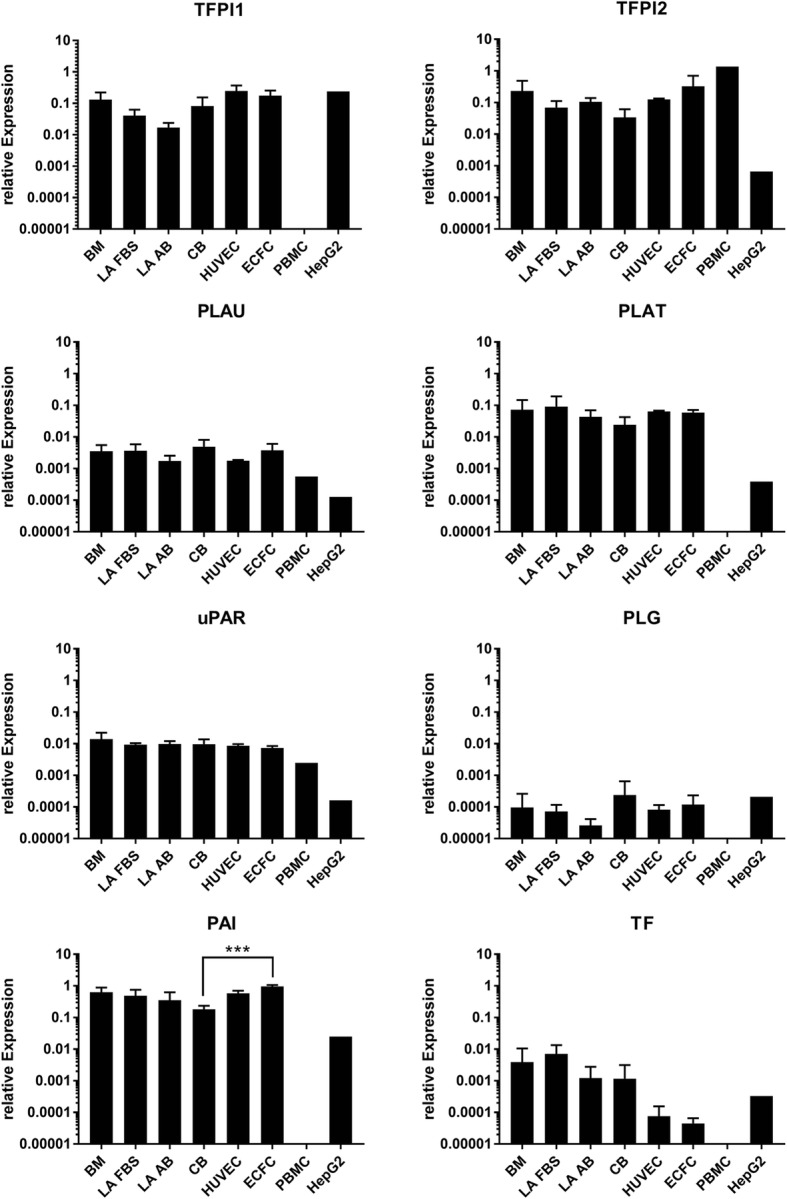
RT-qPCR analysis of prothrombogenic and antithrombogenic genes. Gene expression analyzed in BM *n* = 3, LA FBS *n* = 3, LA human AB-serum *n* = 2, CB *n* = 3, HUVECs *n* = 3, ECFCs *n* = 3, PBMCs, HepG2 cells and HeLa cells each *n* = 1. ***p < 0.001. BM bone marrow, CB cord blood, ECFC endothelial colony forming cell, FBS fetal bovine serum, HUVEC human umbilical vein endothelial cell, LA lipoaspirate, PBMC peripheral blood mononuclear cell

In the present study, we identified CD73-generated adenosine as the major mechanism by which MSCs inhibit platelet activation. Hemostasis, however, involves at least three steps: vasoconstriction, platelet activation/plug formation and coagulation. To estimate the potential involvement in the coagulation cascade and to compare with previously published data [6, 12–14, 16, 19], we finally assessed the gene expression of different procoagulatory and anticoagulatory genes. Comparing MSCs to endothelial cells—HUVECs and cord blood-derived endothelial colony forming cells (CB-ECFC) [22]—no differences were observable, except for TF expression that was apparently higher in all MSCs (Fig. 7). CB-MSCs compared to CB-ECFCs had a reduced expression of plasminogen- activator inhibitor (PAI). Thus, we conclude that the observed source-specific differences in platelet reactivity are based on the identified adenosine metabolism rather than on coagulation.

## Discussion

With respect to the safety of MSC infusion, our paper combines a translationally relevant issue with an important basic research question about the underlying mechanism. Focusing on the effect of MSCs on platelet function, we document that BM-MSCs and LA-MSCs, and with batch variations also CB-MSCs, inhibited the agonist-induced activation and aggregation of platelets—even more than endothelial cells, well known to regulate platelet reactivity. This inhibitory activity was confirmed to happen in both PRP and whole blood by applying a variety of platelet function tests including point-of-care diagnostic tests, underlining the physiological relevance.

**Fig. 8:**
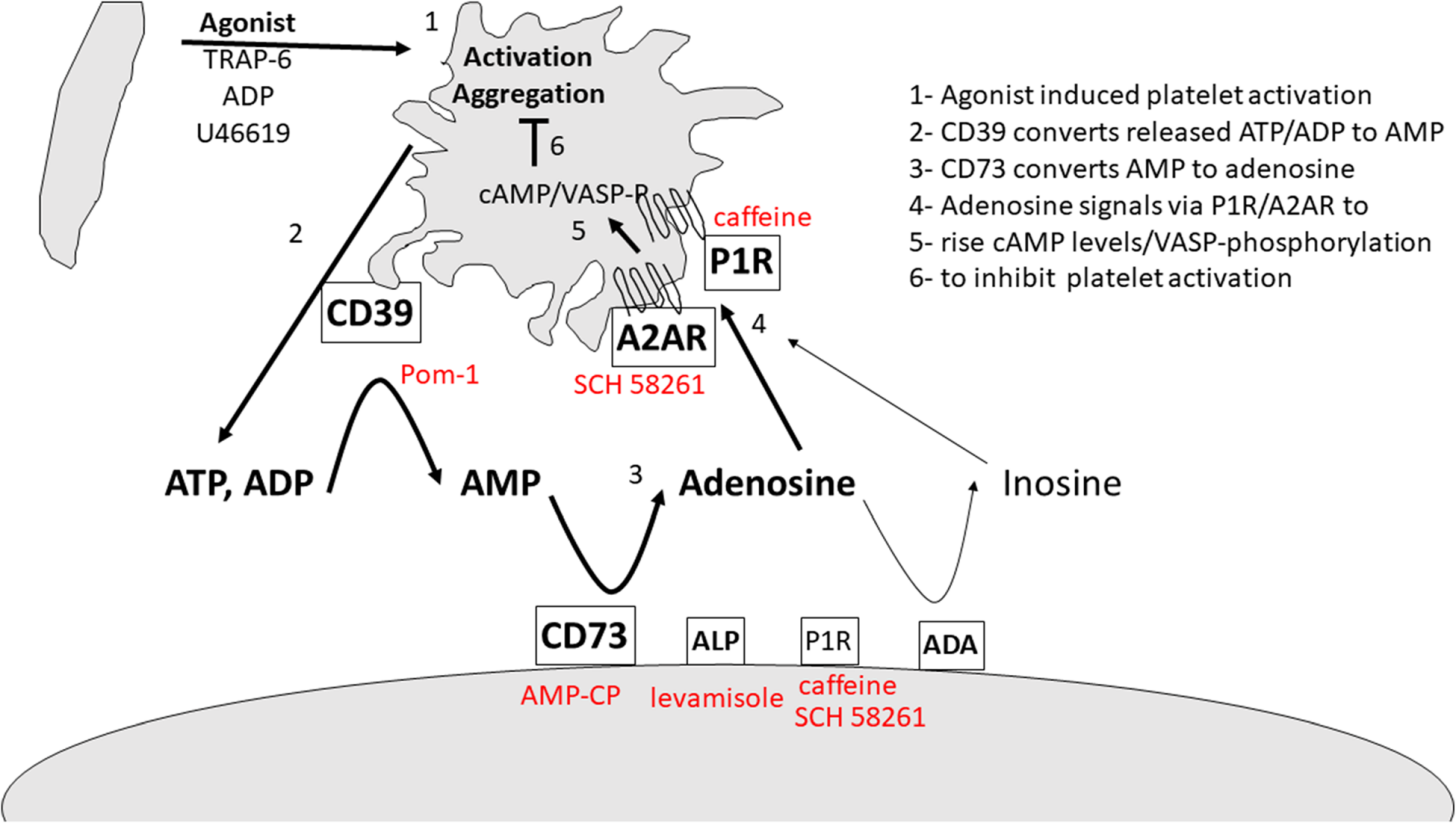
Graphical summary of results. Upon agonist-induced platelet activation, ATP and ADP are released. These are converted to AMP by platelet CD39 activity. AMP is converted to adenosine by MSC-expressed CD73 and to a low extent by alkaline phosphatase. Adenosine signals vial A2AR and other P1 receptors to raise cAMP levels and to induce VASP phosphorylation. This reduces further platelet activation. Used inhibitors indicated in red. ADA adenosine deaminase, ADP adenosine diphosphate, ALP alkaline phosphatase, AMP adenosine monophosphate, ATP adenosine triphosphate, TRAP thrombin receptor activator for peptide, VASP vasodilator-stimulated phosphoprotein

We identified the underlying mechanism to involve CD73-converted adenosine as summarized in Fig. 8. Activated platelets release ATP and ADP from their dense granules. Subsequent dephosphorylation of these agonists to the antagonists AMP by platelet CD39 and adenosine by MSC CD73 induces P1 receptor signaling to raise cAMP levels and VASP phosphorylation. This finally stops the activation cascade and reduces excessive platelet reactivity.

In fact, we confirmed that adenosine is produced in MSC–platelet cocultures at levels inhibitory for platelet function in PRP and whole blood. Using inhibitors of these enzymes and adenosine receptors, we verified the crucial role of CD73-converted adenosine. The CD39 inhibitor POM-1 had only minor effects on phosphate and adenosine release but inhibited platelet activation per se, supporting the CD39 expression and activity in platelets. In contrast, blockade of CD73 by AMP-CP resulted in a compensation of MSC inhibitory effects along with a significant inhibition of AMP hydrolysis to phosphate and adenosine, correlating receptor expression to function. Caffeine, an unspecific adenosine P1 receptor blocker, and SCH 58261, a specific A2AR antagonist, reduced the inhibitory effect of MSCs and adenosine. The fact that SCH 58261 had a stronger compensatory effect on adenosine than caffeine, but minor effects in MSC cocultures, suggests the involvement of further adenosine receptor subtypes, nucleotide processing enzymes or nucleoside transporters [31, 40, 43]. It is, however, beyond the scope of this study to fully dissect the cascade of purinergic signaling.

Recent studies have documented that MSC CD73-converted adenosine contributes to the immunomodulatory capacities of MSCs [37–40]. As in our study, CD39 and CD73 activity may be influenced by the tissue milieu (e.g. in cancer) and may require cooperation between different cell types [37, 40, 46]. We show that platelets express both CD39 and A2AR, but CD73 only weakly. In contrast, MSCs had low CD39 and low A2AR expression but high CD73 expression. The observed differences between BM-MSCs, LA-MSCs and CB-MSCs may relate to differences in nucleotide hydrolysis activity [32]. In contrast to our data, other authors detected CD39 expression in MSCs. Schuler et al. [40, 46] suggest that CD39 expression is largely influenced by the tissue source and activation state. Kerkelä et al. [37] observed CD39 expression only in BM-MSCs, but not in CB-MSCs. LA-MSCs have been tested negative for CD39 [47]. In addition, MSCs from different murine tissues have been shown to differ with respect to ectonucleotidase activity. Murine adipose tissue-derived MSCs had significantly higher ATP hydrolysis capacity than BM-MSCs, although the AMP hydrolysis activity was comparable. The authors concluded that MSCs exert tissue-specific roles in regulating the purinergic system [48]. Besides CD39, ectonucleotide pyrophosphatases/phosphodiesterases (E-NPPs) could be involved in AMP generation, as shown for HeLa cells and HUVECs [49].

Our data indicate that CD73 is the key enzyme involved in antithrombotic adenosine production. Strikingly, the enzymatic activity of HUVECs was low, despite high CD73 expression. Discrepant expression and activity data have been described and may be based on point mutations, splicing alterations or posttranslational modifications [50–53]. Other enzymes such as alkaline phosphatase could be involved in nucleotide metabolism [31]. ALP activity was detected at differing levels in HeLa cells and MSCs (highest in BM-MSCs, lower in LA-MSCs and low in both CB-MSCs and HUVECs). Using levamisole as an ALP inhibitor, the MSC inhibitory activity was reduced. ADA, which degrades adenosine to inosine, was also found to be active in all tested cells, with quite high activity in LA-MSCs.

Documenting that MSCs induce adenosine signaling, we verified that VASP phosphorylation was increased, indicating adenylate cyclase activity and increased cAMP levels by both MSCs and adenosine. Thus, both CD39-mediated ADP removal plus CD73-mediated adenosine production modulates platelet activation as summarized in Fig. 8.

Of high relevance for translation to the clinic, MSCs exerted their inhibitory effects not only in PRP, but also in whole blood. In whole blood, leukocytes and erythrocytes but also soluble enzymes add to purinergic signaling exerting CD39 and CD73 activity and removing adenosine by equlibrative nucleoside transporters, respectively [34, 46]. Our data show that adenosine produced by MSCs can affect platelet activation, despite the presence of the other cells, to an extent measurable in diagnostic point-of-care tests.

We observed a common inhibitory activity of MSCs with similar effects on all markers and agonist stimulations. Only after U46619 stimulation did the inhibitory effect appear to be weaker. It might therefore be possible that the thromboxane-induced pathway of platelet activation is less impaired by MSCs. Another possibility is that U46619 may act directly on MSCs. Two reports indicate that U46619/thromboxane a(2) affects MSC differentiation, migration and proliferation [54, 55].

Importantly, BM-MSCs and LA-MSCs had no effect on resting platelets. This fits data indicating that MSC-seeded nanofibrous scaffolds were protected from platelet adhesion and thrombus formation [21]. Only some batches of CB-MSCs induced activation marker expression in resting platelets, similar to the tested tumor cell line HeLa. The effect of CB-MSCs on resting platelets may indicate an increased thromboembolic risk associated with their application, or in a different setting, a beneficial hemostatic potential [13]. Expression of various prothrombotic and antithrombotic genes, however, was similar for the MSCs from different cellular sources. These data support our previous findings that CB-MSCs differ from BM-MSCs and LA-MSCs in several aspects, namely frequency, differentiation, immunomodulation, cell marker expression and size [23, 34]. Besides intrinsic heterogeneity of MSC preparations, CB appears to generate at least two distinct MSC-like populations [23, 56, 57]. It is a matter of future studies to correlate heterogeneity to function.

Interestingly, conditioned MSC medium had no impact on resting platelets or on the degree of agonist-induced activation, in contrast to previous reports suggesting a releasable ADPase activity in polymorphonuclear leukocytes [58]. We conclude that there is no significant production of any soluble platelet-affecting substances under standard cell culture conditions, but that the inhibitory activity is exerted by cell-bound CD73, which metabolizes extracellular AMP to adenosine.

As our major goal is to ensure the safety of MSC infusions, it is imperative to understand the effects of MSCs on hemostasis after systemic infusion. Hemostasis is a multistep process involving vasoconstriction, platelet plug formation and coagulation, and finally fibrinolysis. MSC involvement has been evaluated previously, focusing on individual steps:

1. MSC conditioned media may promote vasodilation of pulmonary artery rings [59].
2. TF expression by MSCs may cause thromboembolism, preventable by use of, or example, heparin [12, 15].
3. Fibrinolytic activity may regulate migration and wound healing [60, 61].

As a fourth mechanism by which MSCs can influence hemostasis, this study shows that MSCs prevent excessive platelet responsiveness by CD73-converted adenosine.

The strength of this study is the combination of a translationally relevant issue with an important basic research question about the underlying mechanism. Our conclusions build on robust data based on a large number of MSC and platelet combinations, and MSCs from three different tissues plus two control cell types, with the effects demonstrated in both PRP and whole blood and using a variety of different test systems. The work opens some new lines of inquiry and questions to be answered in subsequent studies, including tissue-specific CD73 expression/activity and the interplay of other cell-bound and soluble nucleotide processing factors.

## Conclusions

Our study documents that MSCs do not induce platelet activation and thereby thrombus formation, but rather actively inhibit platelet activation by a CD73 activity generating antithrombotic adenosine. CB-MSCs show batch-dependent differences. Since CD73 activity has been further linked to tissue barrier function, adaptation to ischemic conditions/hypoxia and inflammation, this mechanism may contribute to the tissue-protective mode of action of MSCs.

## Additional files

Additional file 1: **Table S1.** Primer and probes used for RT-qPCR analysis. (DOCX 20 kb)

Additional file 2: **Figure S1.** Effect of MSCs on platelet adhesion and aggregation under shear flow conditions. To assess effect of MSCs on platelet activation under shear flow conditions, we performed microfluidic experiments using a pneumatically driven channel system (BioFlux, San Francisco, CA, USA) mounted on an inverted microscope capable of live cell reflectance interference contrast microscopy (RICM) as described previously [31]. Briefly, channels were coated with 10 μg/cm^2^ fibronectin (from human plasma F2006; Sigma Aldrich, St. Louis, MO, USA). The coated channels were filled with 300 μl of native whole blood with and without 1.5 × 10^5^ BM-MSCs upon hematocrit adjustment and perfused with a constant shear stress of 5 dyne/cm^2^. At indicated points in time, RICM photographs of channel footprints were taken and analyzed by counting the number of adherent/aggregated platelets. BM-MSCs *n* = 3. (TIF 1135 kb)

Additional file 3: **Figure S2.** Effect of MSCs on resting and agonist-induced platelet activation in impedance aggregometry. Impedance aggregometry experiments conducted using Multiplate® analyzer (Roche Diagnostics, Mannheim, Germany) [31]. Before stimulation, hirudinized whole blood samples were preincubated with respective cells or CM for 10 min, a 7-min phase outside the device followed by 3 min incubation in the aggregometer at 36 °C under stirring. Then 3.3 μM ADP or 6.7 μM TRAP-6 was added for platelet stimulation. Aggregation assessed for 6 min and determined as area under the curve (AUC). Whole blood incubated with two different concentrations of MSCs, HUVECs or HeLa cells. Then 5 μM ADP or TRAP-6 was added to stimulate platelets and impedance was measured. AUC values normalized to respective control without cells. *n* = 2–7. (TIF 784 kb)

Additional file 4: **Figure S3.** Effect of MSCs on aggregation and thrombus formation. To assess aggregation and thrombus formation, fluorescence microscopy experiments were performed. To visualize platelets, PRP prestained for 30 min with Calcein-AM (1 μg/ml; Merck, Darmstadt, Germany). PRP with/without respective cells added to 96-well plates. To observe a cell effect on unstimulated cells, phase-contrast and fluorescence pictures taken after 10 min (Axio Imager D1, Zeiss AG, Oberkochen, Germany, with AxioVision software). Platelets then stimulated with 5 μM TRAP-6 for 10 min until taking another series of pictures. Top row, phase contrast; bottom row, fluorescence microscopy. Representative pictures from *n* = 2–3 experiments. **A–D** 5 μM TRAP-6-stimulated platelets. **A** Platelets w/o other cells. Strong aggregation and thrombus formation visible. **B** Platelets and HeLa cells. Strong aggregation visible with fewer but bigger clots compared to platelets alone. **C** Platelets and CB-MSCs. Small aggregates with many single platelets visible. **D** Platelets and BM-MSCs. No aggregation visible. **E–H** Resting platelets. **E** Platelets w/o other cells. No aggregation visible. **F** Platelets and HeLa cells. Strong platelet aggregation and clotting visible. However, no platelet/HeLa aggregates formed. **G** Platelets and CB-MSCs. No clotting appears, but platelets appear to have undergone morphological changes indicating activation and adhesion. **H** Platelets and BM-MSCs. Single platelets grouped around BM-MSCs without aggregation or any evidence for activation. **I** Platelet aggregate size. Photomicrographs in **A–H** analyzed with respect to aggregate size using ImageJ (*n* = 3 biological replicates for MSCs and HUVECs, *n* = 3 technical replicates for HeLa, different fields of vision analyzed). **J** Platelet binding to MSCs. Cells gated on FSC/SSC and assessed for CD41 positivity indicative of platelet binding. No CD41 positivity detectable in cocultures with unstimulated and stimulated platelets. Representative histograms, mean ± SD CD41 positivity values given relative to w/o platelet control from *n* = 3 experiments (technical replicates for HeLa). (TIF 8302 kb)

Additional file 5: **Figure S4.** Effect of MSCs on platelet activation using different agonists and pathway inhibitors. **A, B** Effect of 10^5^ LA-MSCs/ml on platelet activation after stimulation with different agonists ADP, TRAP-6 and U46619 (*n* = 4). Expression of two different activation markers shown: **A** CD62P and **B** PAC-1 binding. *p < 0.05. **C, D** Effect of AK4 and indomethacin on platelet inhibition by 5 × 10^5^ BM-MSCs/ml. Platelets stimulated with TRAP-6. *x* axis, PAC-1 fluorescence intensity; *y*, axis, platelet count. One of two experiments shown: **C** AK4 to block CD62P and **D** MSC preculture in indomethacin to block COX. (TIF 176 kb)

## Abbreviations

A2AR: Adenosine 2A receptor
ADA: Adenosine deaminase
ADP: Adenosine diphosphate
ALP: Alkaline phosphatase
AMP: Adenosine monophosphate
AMP-CP: α,β-Methyleneadenosine 5′-diphosphate
ATP: Adenosine triphosphate
BM: Bone marrow
CB: Cord blood
CM: Conditioned medium
COX: Cyclooxygenase
CPDA: Citrate phosphate dextrose adenine
ESI: Electrospray ionization
FBS: Fetal bovine serum
HUVEC: Human umbilical vein endothelial cell
IBMIR: Instant blood-mediated inflammatory reaction
LA: Lipoaspirate
MFI: Mean fluorescence intensity
MRM: Multiple reaction monitoring
MSC: Mesenchymal stromal cell
PAR-1: Protease-activated receptor 1
PFA: Platelet function analyzer
POM-1: Sodium polyoxotungstate
RT-qPCR: Real-time quantitative PCR
TF: Tissue factor
TRAP-6: Thrombin receptor activator for peptide 6
VASP: Vasodilator-stimulated phosphoprotein
