# Supplementary material for: Human mesenchymal stromal cells inhibit platelet activation and aggregation involving CD73-converted adenosine
Source: Stem Cell Res Ther. 2018 Jul 4;9:184. doi: 10.1186/s13287-018-0936-8 (PMC6033237; doi:10.1186/s13287-018-0936-8)
Supplement: Supplementary file 1 — Table S1. Primer and probes used for RT-qPCR analysis. (DOCX 20 kb) [file 13287_2018_936_MOESM1_ESM.docx]

## Table S1: Primes and probes used for RT-qPCR analysis

|  | **Gene** | **Accession No** | **UPL** | **Sequence** | **Position** | **Intron** | **Amplicon** |
| --- | --- | --- | --- | --- | --- | --- | --- |
| **TFPI** | Homo sapiens tissue factor pathway inhibitor (lipoproteinassociated coagulation inhibitor), transcript variant 1, mRNA | NM_006287.4 | 61 | tgaacgtttcaagtatggtgga | 745 - 766 | 594 | 114 |
|  |  |  |  | ccataattatccacctggaaacc | 836 - 858 |  |  |
| **TFPI2** | Homo sapiens tissue factor pathway inhibitor 2, mRNA | NM_006528.2 | 51 | tctgctgcttttcctgacg | 108 - 126 | 267 | 132 |
|  |  |  |  | tcgtagtagtaacggagaagtaggg | 215 - 239 |  |  |
| **TF** | Homo sapiens coagulation factor III (thromboplastin, tissue factor), transcript variant 1, mRNA | NM_001993.4 | 51 | ggagaaaggggaattcagaga | 953 - 973 | 1722 | 140 |
|  |  |  |  | gggagttctccttccagctc | 1073 - 1092 |  |  |
| **PAI** | Homo sapiens serpin peptidase inhibitor, clade E (nexin, plasminogen activator inhibitor type 1), member 1, mRNA | NM_000602.2 | 80 | ctcctggttctgcccaagt | 1031 - 1049 | 1598 | 66 |
|  |  |  |  | caggttctctaggggcttcc | 1077 - 1096 |  |  |
| **PLG** | Homo sapiens plasminogen, mRNA | NM_000301.2 | 22 | aacaagcgctgggaacttt | 814 - 832 | 1530 | 74 |
|  |  |  |  | cactggtaggtgggaccaga | 868 - 887 |  |  |
| **PLAU** | Homo sapiens plasminogen activator, urokinase , transcript variant 1, mRNA | NM_002658.3 | 46 | ttgctcaccacaacgacatt | 955 - 974 | 646 | 94 |
|  |  |  |  | ggcaggcagatggtctgtat | 1029 - 1048 |  |  |
| **uPAR** | Homo sapiens plasminogen activator, urokinase receptor, transcript variant 1, mRNA | NM_002659.2 | 22 | cctctgcaggaccacgat | 362 - 379 | 2119 | 89 |
|  |  |  |  | tggtcttctctgagtgggtaca | 429 - 450 |  |  |
| **PLAT** | Homo sapiens plasminogen activator, tissue, transcript variant 1, mRNA | NM_000930.3 | 15 | gctgacgtgggagtactgtg | 1064 - 1083 | 1251 | 70 |
|  |  |  |  | ctgaggctggctgtactgtct | 1113 - 1133 |  |  |
| **GAPDH** | glycerinaldehyd-3-phosphat dehydrogenase | NM_002046.3 | 45 | tccactggcgtcttcacc | 394 - 411 | 88 | 78 |
|  |  |  |  | ggcagagatgatgaccctttt | 451 - 471 |  |  |
| **SFRS4** | Splicing-Factor 4 | NM_005626.3 | 51 | agctggcaagacctaaagga | 443 - 462 | 4460 | 68 |
|  |  |  |  | cccttgtgagcatctgcat | 492 - 510 |  |  |
