# Supplementary figures and images for: Human mesenchymal stromal cells inhibit platelet activation and aggregation involving CD73-converted adenosine
Source: Stem Cell Res Ther. 2018 Jul 4;9:184. doi: 10.1186/s13287-018-0936-8 (PMC6033237; doi:10.1186/s13287-018-0936-8)

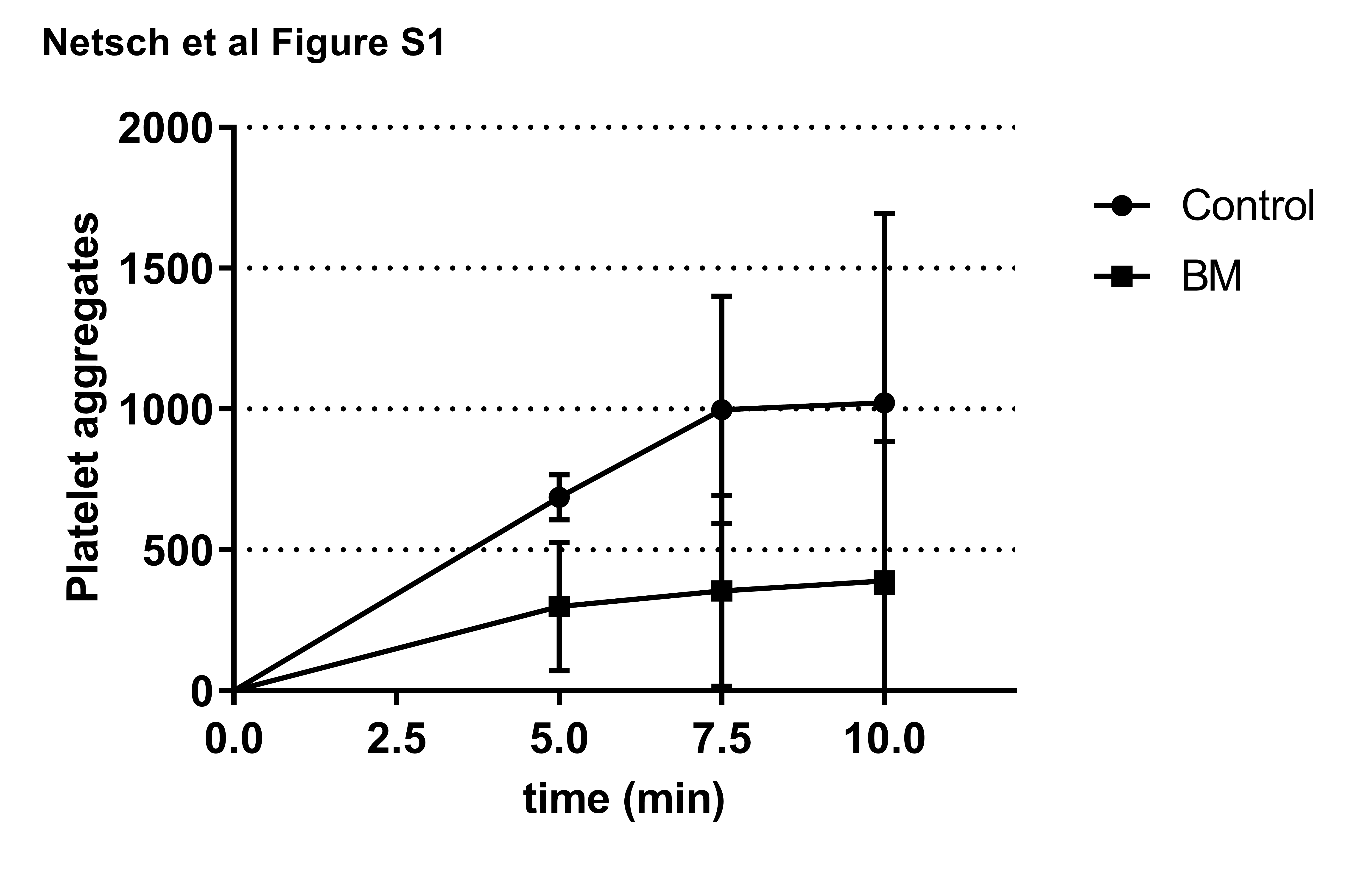

Supplement: Supplementary file 2 — Figure S1. Effect of MSCs on platelet adhesion and aggregation under shear flow conditions. To assess effect of MSCs on platelet activation under shear flow conditions, we performed microfluidic experiments using a pneumatically driven channel system (BioFlux, San Francisco, CA, USA) mounted on an inverted microscope capable of live cell reflectance interference contrast microscopy (RICM) as described previously [31]. Briefly, channels were coated with 10 μg/cm2 fibronectin (from human plasma F2006; Sigma Aldrich, St. Louis, MO, USA). The coated channels were filled with 300 μl of native whole blood with and without 1.5 × 10^5^ BM-MSCs upon hematocrit adjustment and perfused with a constant shear stress of 5 dyne/cm2. At indicated points in time, RICM photographs of channel footprints were taken and analyzed by counting the number of adherent/aggregated platelets. BM-MSCs n = 3. (TIF 1135 kb) [file 13287_2018_936_MOESM2_ESM.tif]

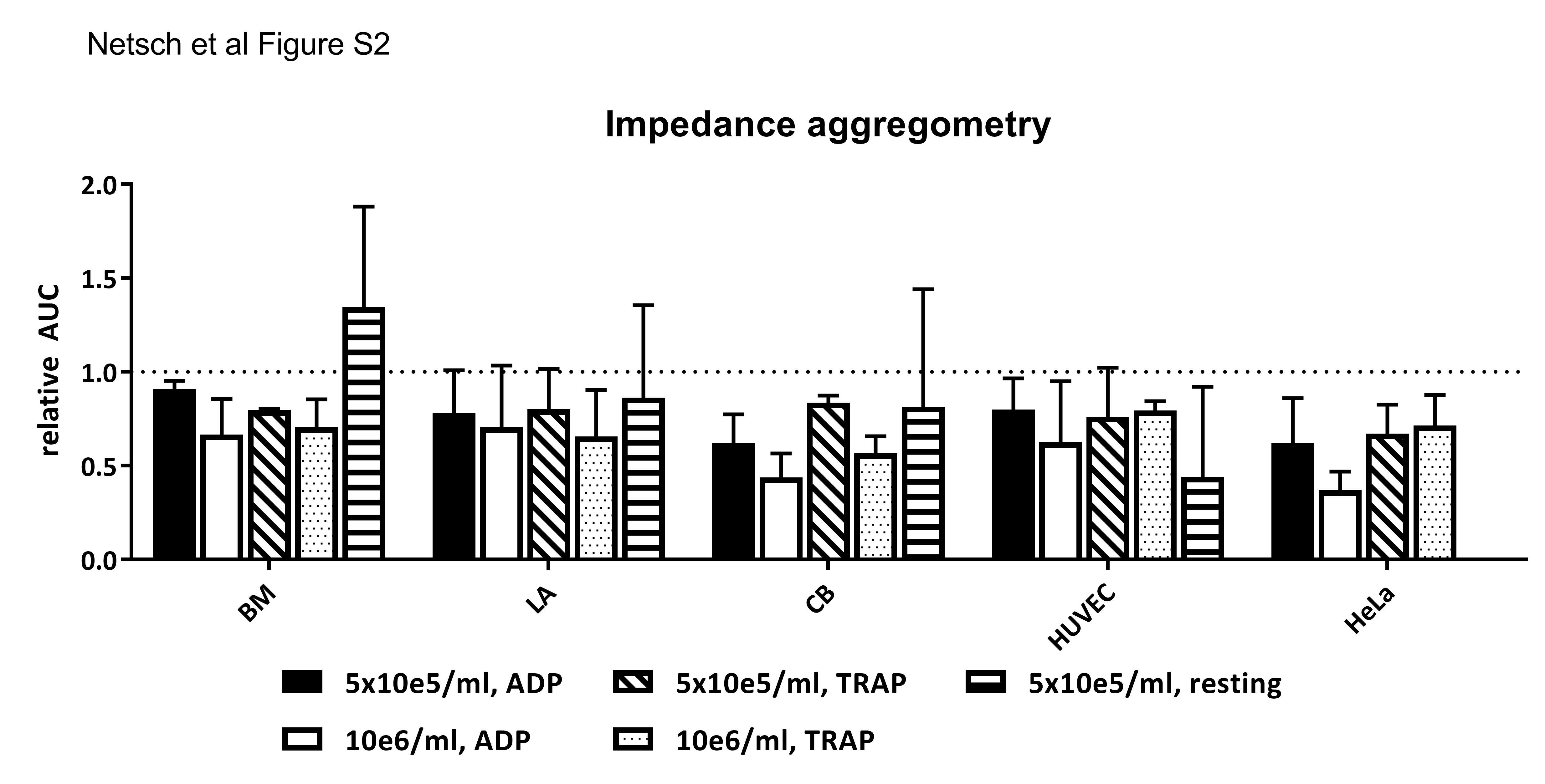

Supplement: Supplementary file 3 — Figure S2. Effect of MSCs on resting and agonist-induced platelet activation in impedance aggregometry. Impedance aggregometry experiments conducted using Multiplate® analyzer (Roche Diagnostics, Mannheim, Germany) [31]. Before stimulation, hirudinized whole blood samples were preincubated with respective cells or CM for 10 min, a 7-min phase outside the device followed by 3 min incubation in the aggregometer at 36 °C under stirring. Then 3.3 μM ADP or 6.7 μM TRAP-6 was added for platelet stimulation. Aggregation assessed for 6 min and determined as area under the curve (AUC). Whole blood incubated with two different concentrations of MSCs, HUVECs or HeLa cells. Then 5 μM ADP or TRAP-6 was added to stimulate platelets and impedance was measured. AUC values normalized to respective control without cells. n = 2–7. (TIF 784 kb) [file 13287_2018_936_MOESM3_ESM.tif]

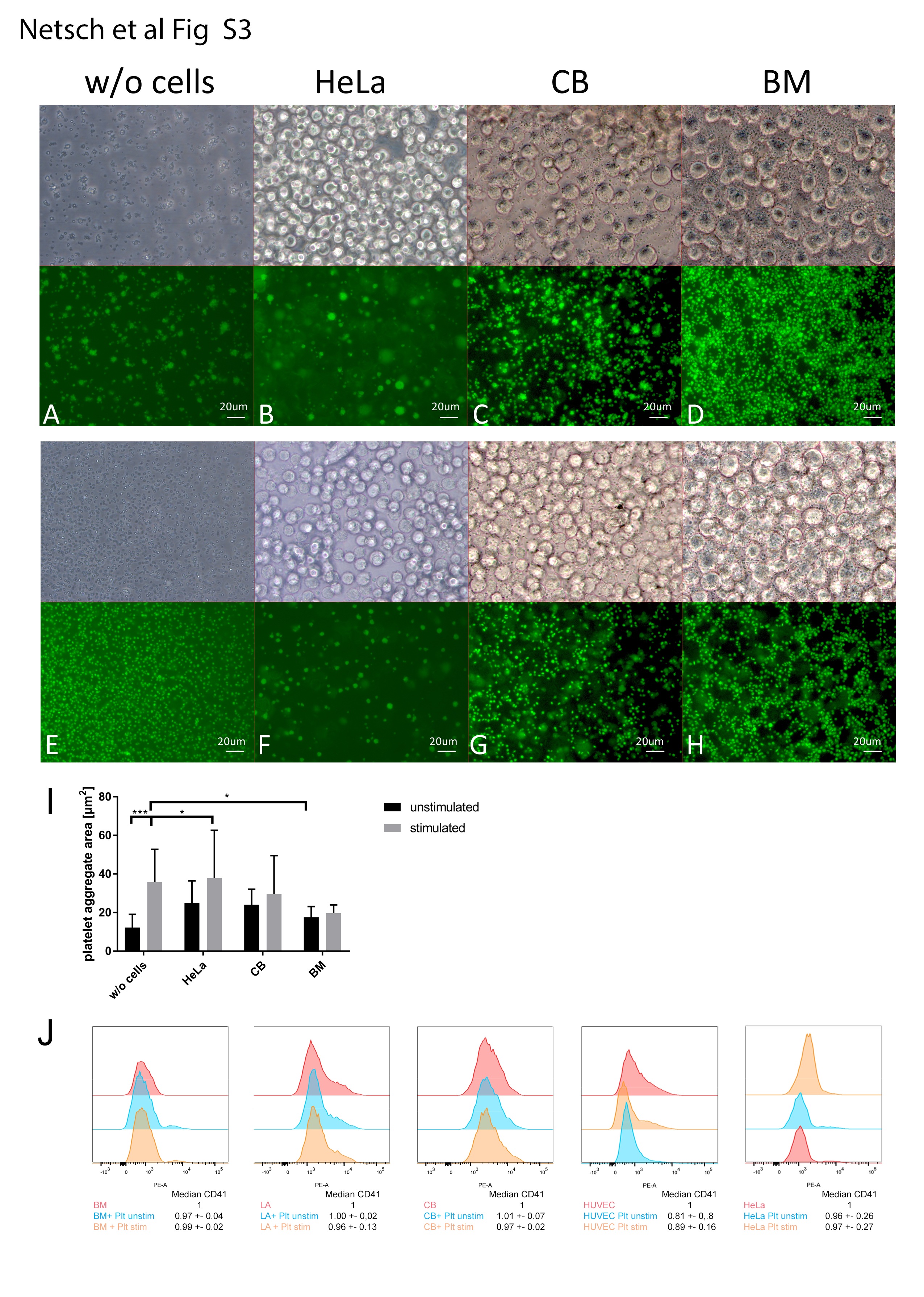

Supplement: Supplementary file 4 — Figure S3. Effect of MSCs on aggregation and thrombus formation. To assess aggregation and thrombus formation, fluorescence microscopy experiments were performed. To visualize platelets, PRP prestained for 30 min with Calcein-AM (1 μg/ml; Merck, Darmstadt, Germany). PRP with/without respective cells added to 96-well plates. To observe a cell effect on unstimulated cells, phase-contrast and fluorescence pictures taken after 10 min (Axio Imager D1, Zeiss AG, Oberkochen, Germany, with AxioVision software). Platelets then stimulated with 5 μM TRAP-6 for 10 min until taking another series of pictures. Top row, phase contrast; bottom row, fluorescence microscopy. Representative pictures from n = 2–3 experiments. A–D 5 μM TRAP-6-stimulated platelets. A Platelets w/o other cells. Strong aggregation and thrombus formation visible. B Platelets and HeLa cells. Strong aggregation visible with fewer but bigger clots compared to platelets alone. C Platelets and CB-MSCs. Small aggregates with many single platelets visible. D Platelets and BM-MSCs. No aggregation visible. E–H Resting platelets. E Platelets w/o other cells. No aggregation visible. F Platelets and HeLa cells. Strong platelet aggregation and clotting visible. However, no platelet/HeLa aggregates formed. G Platelets and CB-MSCs. No clotting appears, but platelets appear to have undergone morphological changes indicating activation and adhesion. H Platelets and BM-MSCs. Single platelets grouped around BM-MSCs without aggregation or any evidence for activation. I Platelet aggregate size. Photomicrographs in A–H analyzed with respect to aggregate size using ImageJ (n = 3 biological replicates for MSCs and HUVECs, n = 3 technical replicates for HeLa, different fields of vision analyzed). J Platelet binding to MSCs. Cells gated on FSC/SSC and assessed for CD41 positivity indicative of platelet binding. No CD41 positivity detectable in cocultures with unstimulated and stimulated platelets. Representative histograms, mean ± S [file 13287_2018_936_MOESM4_ESM.tif]

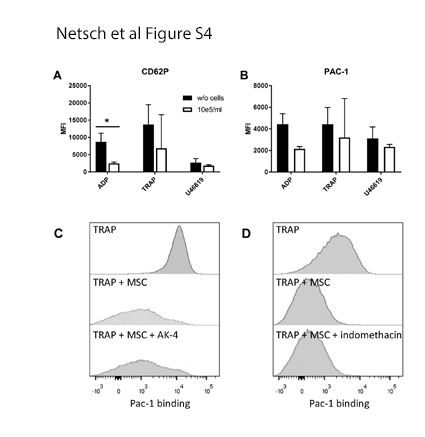

Supplement: Supplementary file 5 — Figure S4. Effect of MSCs on platelet activation using different agonists and pathway inhibitors. A, B Effect of 105 LA-MSCs/ml on platelet activation after stimulation with different agonists ADP, TRAP-6 and U46619 (n = 4). Expression of two different activation markers shown: A CD62P and B PAC-1 binding. *p < 0.05. C, D Effect of AK4 and indomethacin on platelet inhibition by 5 × 10^5^ BM-MSCs/ml. Platelets stimulated with TRAP-6. x axis, PAC-1 fluorescence intensity; y, axis, platelet count. One of two experiments shown: C AK4 to block CD62P and D MSC preculture in indomethacin to block COX. (TIF 176 kb) [file 13287_2018_936_MOESM5_ESM.tif]
